# The neuroprotective effects of SIRT1 in mice carrying the APP/PS1 double-transgenic mutation and in SH-SY5Y cells over-expressing human APP670/671 may involve elevated levels of α7 nicotinic acetylcholine receptors

**DOI:** 10.18632/aging.102713

**Published:** 2020-01-30

**Authors:** Kun Cao, Yang-Ting Dong, Jie Xiang, Yi Xu, Yi Li, Hui Song, Wen-Feng Yu, Xiao-Lan Qi, Zhi-Zhong Guan

**Affiliations:** 1 Department of Pathology at Guizhou Medical University and Pathology Department in Affiliated Hospital of Guizhou Medical University, Guiyang, Guizhou, P. R. of China; 2 Key Laboratory of Endemic and Ethnic Diseases of the Ministry of Education of P. R. China (Guizhou Medical University), Guiyang, Guizhou, P. R. of China; 3 Key Laboratory of Medical Molecular Biology, Guiyang, Guizhou, P. R. of China

**Keywords:** Alzheimer’s disease, APP/PS1 mice, SIRT1, α7 nAChR, MAPK

## Abstract

The aim was to determine whether the neuroprotective effect of SIRT1 in Alzheimer’s disease (AD), due to inhibition of aggregation of the β-amyloid peptide (Aβ), involves activation of α7 nAChR. In present study, four-month-old APP/PS1 mice were administered resveratrol (RSV) or suramin once daily for two months, following which their spatial learning and memory were assessed using the Morris water maze test. Deposits of Aβ in vivo were detected by near-infrared imaging (NIRI) and confocal laser scanning. SH-SY5Y/APP_swe_ cells were treated with RSV, suramin, U0126 or methyllycaconitine (MLA). Levels of proteins and mRNA were determined by Western blotting and qRT-PCR, respectively. The results show that activation of SIRT1 improved their spatial learning and memory and reduced the production and aggregation of Aβ in the hippocampus and cerebral cortex; whereas inhibition of SIRT1 had the opposite effects. In addition, activation of SIRT1 increased the levels of both α7 nAChR and αAPP in the brains these animals. Finally, activation of SIRT1 elevated the levels of pERK1/2, while inhibition of ERK1/2 counteracted the increase in α7 nAChR caused by RSV. These findings indicate that neuroprotection by SIRT1 may involve increasing levels of α7 nAChR through activation of the MAPK/ERK1/2 signaling pathway.

## INTRODUCTION

Alzheimer’s disease (AD) currently afflicts more than 35 million people worldwide [1] and the Delphi study predicted that this number will rise to 42.3 million in 2020 and 81.1 million in 2040 [2]. This neurodegenerative disease is characterized by a number of neuropathological changes, including deposits of β-amyloid peptides (Aβ), neurofibrillary tangles, and large-scale loss of neuron [3]. Accumulating evidence indicates that Aβ, hyperphosphorylated Tau protein, abnormal expression of nicotinic acetylcholine receptors, oxidative stress and inflammation are associated with the pathogenesis of AD [4–7]. In addition, the amyloid cascade hypothesis is supported by extensive experimental findings showing that aggregation of Aβ into fibrils and/or other self-assembling states is central to this process. However, the failure of recent clinical anti-amyloidgenic trials has again raised questions concerning the involvement of this cascade [8–10]. Thus, an improved understanding of the molecular mechanisms underlying AD is necessary for the development of novel, more effective strategies for diagnosis and treatment.

Sirtuins, an evolutionarily conserved family of nicotinamide adenine dinucleotide (NAD)-dependent histone/protein deacetylases, are implicated in a variety of cellular functions ranging from gene silencing and cell cycle regulation to energy homeostasis [11–13]. Among the seven mammalian sirtuins (referred to as SIRT1-7), SIRT1 has been most extensively investigated and is proposed to be involved in a variety of human diseases, including diabetes, cancer and cardiovascular disorders [14–16]. In addition, SIRT1 protects against neuroprotective disorders, including AD [17–18].

Some studies indicate that SIRT1 protects against formation of Aβ and oxidative stress [19–20]. Furthermore, by regulating the activity of several protein substrates, including p53 and peroxisome proliferator-activated receptor-gamma coactivator 1α (PGC-1α) [21], SIRT1 reduces accumulation of Aβ and improves mitochondrial function [22]. Recent research also shows that activation of SIRT1 protects neurons against Aβ1-42-induced disruption of spatial learning, memory, and synaptic plasticity and counteracts the reduction of SIRT1 expression in hippocampus of rats [23]. Moreover, our own findings reveal that activation of SIRT1 attenuates the oxidative stress caused by amyloid-peptide [24]. These observations identify SIRT1 as a promising therapeutic target for overcoming neurodegeneration.

The nicotinic acetylcholine receptor (nAChR), a number of the family of pentameric ligand-gated ion channels, contains 12 subunits designated α2-α10 and β2-β4. (α4)_2_(β2)_3_ and (α7)_5_ are the major types of nAChRs and compared to other nAChRs, (α7)_5_ is more permeable to Ca^2+^ and Na^+^ upon binding acetylcholine or nicotine [25]. α7 nAChR plays important roles in modulating the release of excitatory neurotransmitters, improving learning and memory, and enhancing cognitive function.

Importantly, the expression and function of α7 nAChR in the brain of patients with AD and animal models are offered, suggesting that this subtype participates in the pathogenesis of AD [26]. In addition, we previously found that in the hippocampus of patients with AD, the level of α7 nAChR is reduced [27], while expression of this subunit by astrocytes is elevated [28]. Furthermore, we have shown that lovastatin protects against the neurotoxic effects of Aβ on cultured neurons by enhancing the expression of α7 nAChR [29]. Recently, we also observed that activation of α7 nAChR suppresses Aβ aggregation by up-regulating endogenous αB-crystallin via the PI3K/Akt signaling pathway [30].

Accordingly, both SIRT1 and α7 nAChR appear to play important roles in the pathogenesis of AD, but potential interactions between them remain unclear. The current study was designed to characterize their neuroprotective effects with respect to APP metabolism and accumulation of Aβ as well as the underlying mechanism. Our results demonstrate that the neuroprotection afforded by SIRT1 may involve increasing expression of α7 nAChR, by activating the MAPK/ERK1/2 signaling pathway.

## RESULTS

### Spatial learning and memory

**Figure 1:**
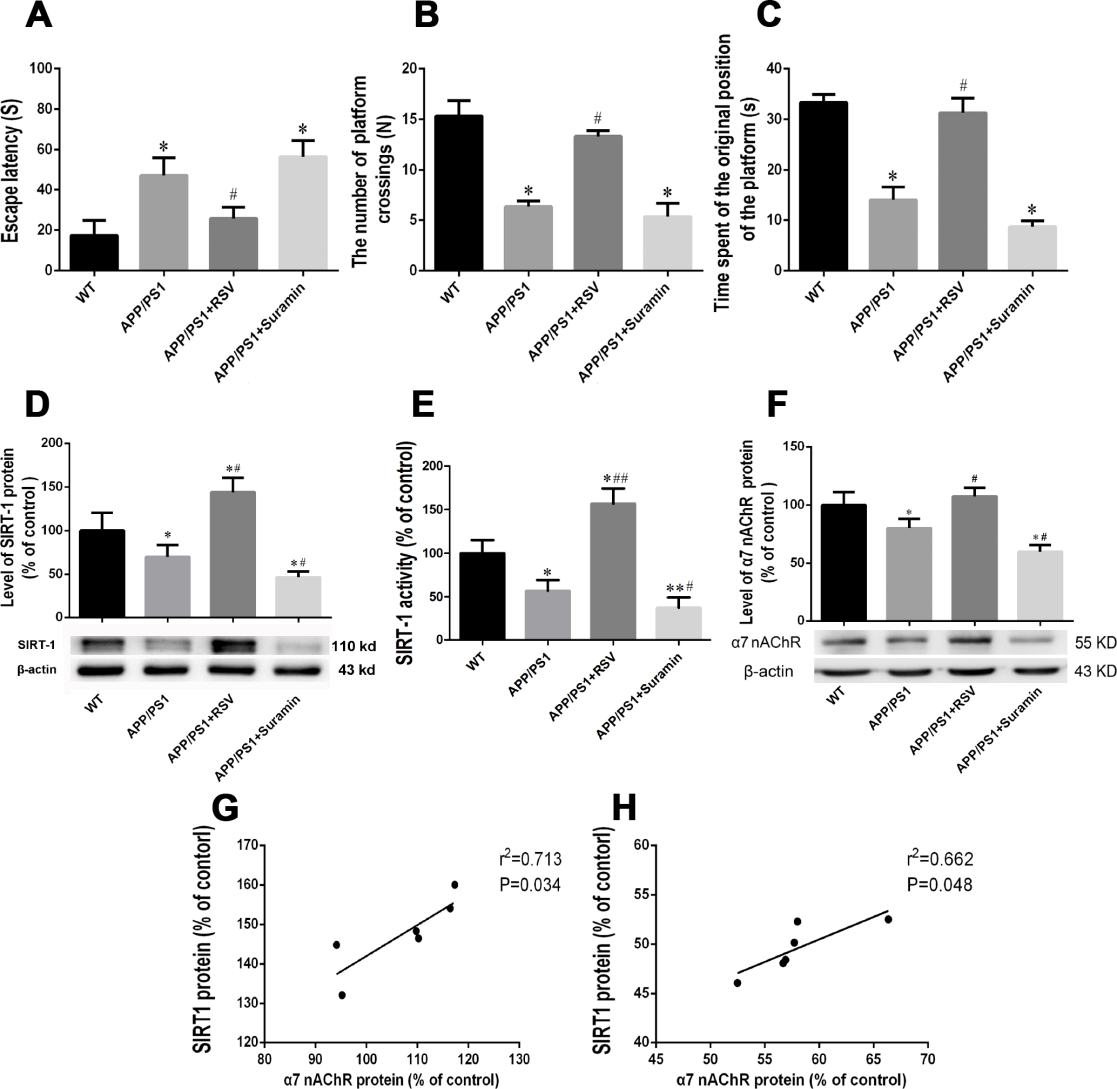
**Effects of an activator and inhibitor of SIRT1 on learning and memory, expression and activity of SIRT1, and the expression of α7 nAChR in mice carrying the APP/PS1 double mutation.** The wild-type (WT) animals received physiological saline (PS) and APP/PS1 mice RSV (20 mg/kg) or suramin (20 mg/kg) by gavage once daily for two months. (**A**) Escape latency. (**B**) The numbers of platform crossings (N). (**C**) Time spent at the original position of platform. (**D**) Relative expression of SIRT1 protein in brain tissue, as determined by Western blotting. (**E**) SIRT1 activity in the brain tissue. (**G**) Correlation between the levels of SIRT1 and α7 nAChR in mice carrying the APP/PS1 double mutation and treated with RSV. (**H**) Correlation between the levels of SIRT1 and α7 nAChR in mice carrying the APP/PS1 double mutation and treated with suramin. The values presented are means ± SD. *P<0.05 compared with the WT group; ^#^*P*<0.05 compared with the APP/PS1 group, as determined by analysis of variance (ANOVA), followed by the Tukey HSD test. Representative western blots are shown beneath **D** and **F**.

After two months of treatment with RSV or Suramin, the escape latency, number of platform crossings and time spent at the original position of the platforms in the Morris water maze differed between the groups of mice. Compared to the WT group, the APP/PS1 group demonstrated longer escape latency, fewer platform crossings and less time at the original position of the platforms (Figure 1A–1C). RSV treatment reduced escape latency and increased both the number of platform crossings and time spent at the original position of the platform in comparison to the APP/PS1 group, and also caused significant changes in all three of these parameters.

### Effects of RSV and Suramin on the expression and activity of SIRT1, as well as the expression of α7 nAChR in mice carrying the APP/PS1 double mutation

Compared to the APP/PS1 group, RSV treatment increased both the expression and activity of SIRT1, as well as expression of α7 nAChR; whereas suramin reduced all three of these parameters (Figure 1D–1F).

### Correlations between the levels of SIRT1 and α7 nAChR

The levels of SIRT1 and α7 nAChR were positively correlated in the mice treat with either RSV (Figure 1G) or suramin (Figure 1H).

### Effects of RSV and suramin on the synaptic proteins, GFAP, Iba-1 and CREB in mice carrying the APP/PS1 double mutation

**Figure 2:**
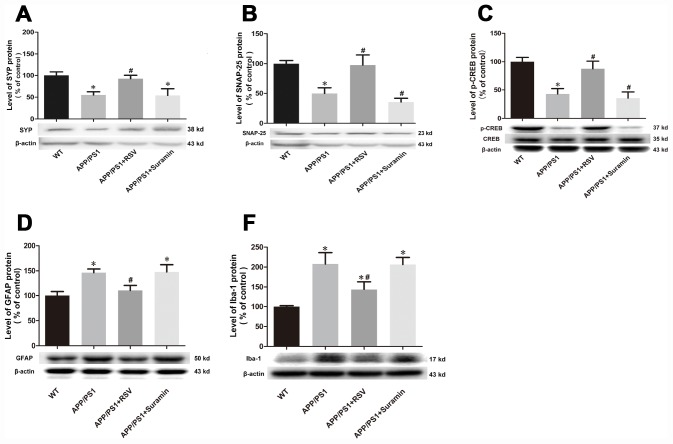
**Effects of RSV and suramin on the expression of synapse makers, p-CREB, GFAP as well as Iba-1, in mice carrying the APP/PS1 double mutation.** (**A**) the level of SYP protein; (**B**) SNAP-25; (**C**) p-CREB; (**D**) GFAP and (**E**) Iba-1. The values presented are means ± SD. *P<0.05 compared with the WT group; ^#^*P*<0.05 compared with the APP/PS1 group, as determined by analysis of variance (ANOVA), followed by the Tukey HSD test. Representative western blots are shown beneath **D** and **F**. Representative western blots are shown beneath each bar graph.

Two synaptic proteins, SYP and SNAP-25, were increased in the brains of mice exposed to RSV and while these levels were reduced in the brains of mice exposed to suramin (Figure 2A, 2B). The level of p-CREB showed a similar change, but CREB did not differ significantly between the different groups of mice (Figure 2C). Furthermore, the levels of GFAP and Iba-1 were reduced in the brains of mice exposed to RSV and while these levels were increased in these animals exposed to suramin (Figure 2D, 2E).

### Effects of RSV and suramin on the Aβ production in mice carrying the APP/PS1 double mutation

**Figure 3:**
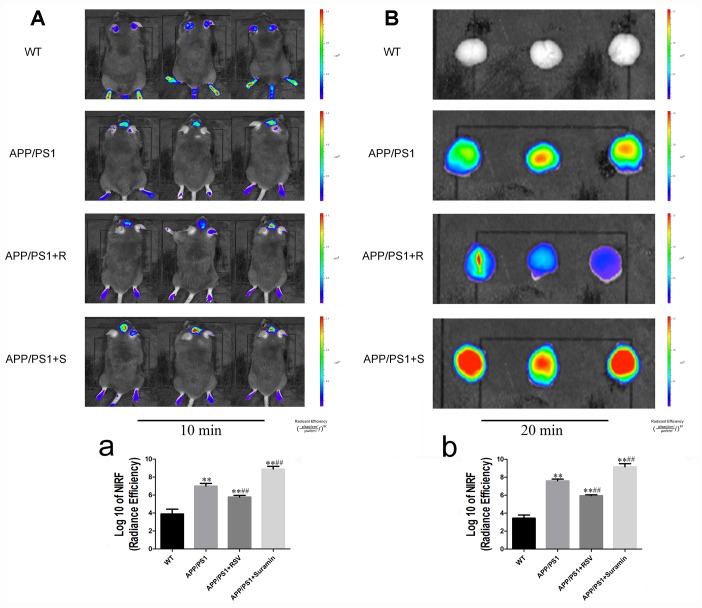
**In vivo and in vitro imaging of in mice carrying the APP/PS1 double mutation at different time-points after intravenous (i.v.) injection of the Aβ probe (CRANAD-58).** The wild-type (WT) animals received physiological saline (PS) and the APP/PS1 mice RSV (APP/PS1+R): 20 mg/kg or suramin (APP/PS1+S): 20 mg/kg by gavage once daily for two months. (**A**) Representative images of 10 min after injection of the probe. (**a**) Quantitation of the fluorescent signals in (**A**). (**B**) Representative images of 20 min after injection of the probe. (**b**) Quantitation analysis of the fluorescent signals in (**B**). Fluorescent signals were detected with excitation at 630 nm and emission at 750 nm. The values presented are means ± SD. *P<0.05 and **P<0.01 compared to the WT group; ^#^*P*<0.05 and ^##^*P*<0.01 compared to the APP/PS1 group.

**Figure 4:**
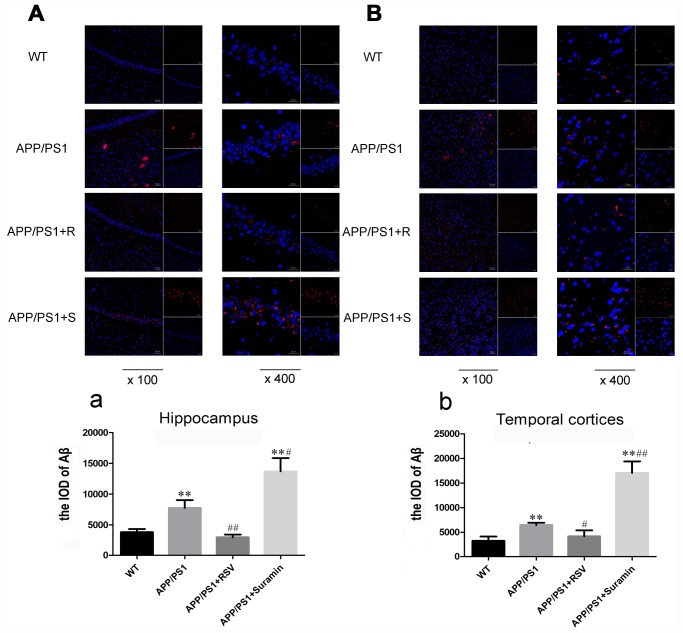
**Effects of RSV and suramin on the level of Aβ in mouse brain, as determined by confocal laser scanning.** The wild-type (WT) animals received physiological saline (PS) and APP/PS1 mice RSV (APP/PS1+R): 20 mg/kg or suramin (APP/PS1+S): 20 mg/kg by gavage once daily for two months. (**A**) Aβ deposits in the hippocampus (×100 and ×400). (**a**) The integral optical density (IOD) of Aβ in the hippocampus. (**B**) Aβ deposits in the temporal cortices (×100 and ×400). (**b**) The IOD of Aβ in the temporal cortices. The values presented are means ± SD. *P<0.05 and **P<0.01 compared to the WT group; ^#^ *P*<0.05 and ^##^*P*<0.01 compared to the APP/PS1 group, as determined by analysis of variance (ANOVA), followed by the Tukey HSD test.

Near-infrared imaging in vivo revealed clearly visible deposits of Aβ in the APP/PS1 and suramin-treated animals (Figure 3A and 3B). Following RSV treatment, the level of these deposits was reduced remarkably (Figure 3a and 3b). In contrast, there were more Aβ plaques in both the hippocampus and temporal cortices of suramin-treated than APP/PS1 mice (Figure 4A/a and 4B/b). These observations indicate that activation of SIRT1 might alleviate nerve damage caused by deposition of Aβ.

### Effects of RSV and Suramin on the expression and activity of SIRT1, as well as the expression of αAPP and α7 nAChR in SH-SY5Y/APPswe cells

**Figure 5:**
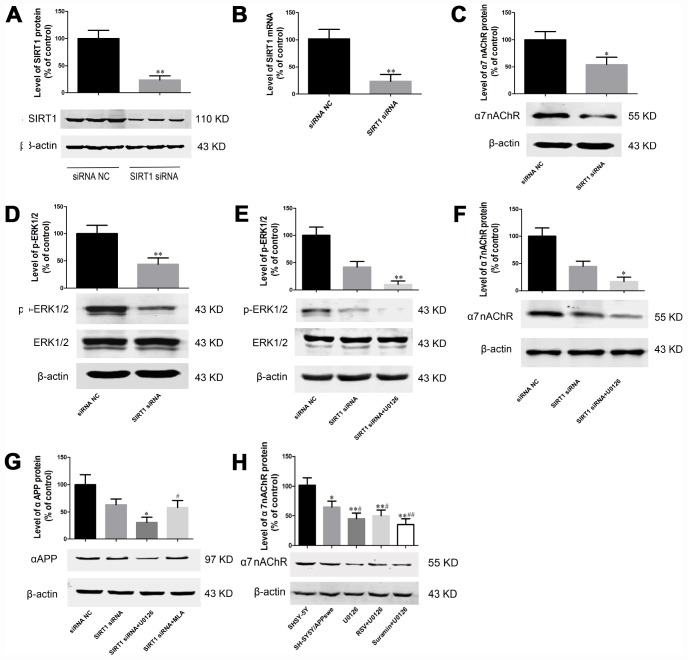
**Effects of RSV and suramin on the expression of αAPP, α7 nAChR and SIRT1, as well as SIRT1 activating in SH-SY5Y/APPswe cells.** The cells were treated with 50μM RSV or 300 μg/ml suramin for 24 h. (**A**) SIRT1 expression. (**B**) SIRT1 activity. (**C**) αAPP expression. (**D**) α7 nAChR expression. The values presented are means ± SD. *P<0.05 and **P<0.01 compared with untreated cells, ^#^*P*<0.05 and ^##^*P*<0.01 compared to the untreated control cells, as determined by analysis of variance (ANOVA), followed by the Tukey HSD test. Representative western blots are shown beneath **A**, **C** and **D**.

To explore the mechanism underlying reduction of Aβ deposition by activation of SIRT1, SH-SY5Y/APPswe cells were exposed to RSV or suramin for 24 h. Consistent with the in vivo findings, RSV increased both the expression and activity of SIRT1, while sruamin exhibited opposite effects (Figure 5A and 5B). In addition, western blotting revealed that activation of SIRT1 elevated expression of αAPP and α7 nAChR, while inhibition of SIRT1 reduced the levels of these proteins (Figure 5C and 5D).

### Effects of SIRT1 siRNA on the expression of α7 nAChR, p-ERK1/2, and ERK1/2 by SH-SY5Y/swe cells

**Figure 6:**
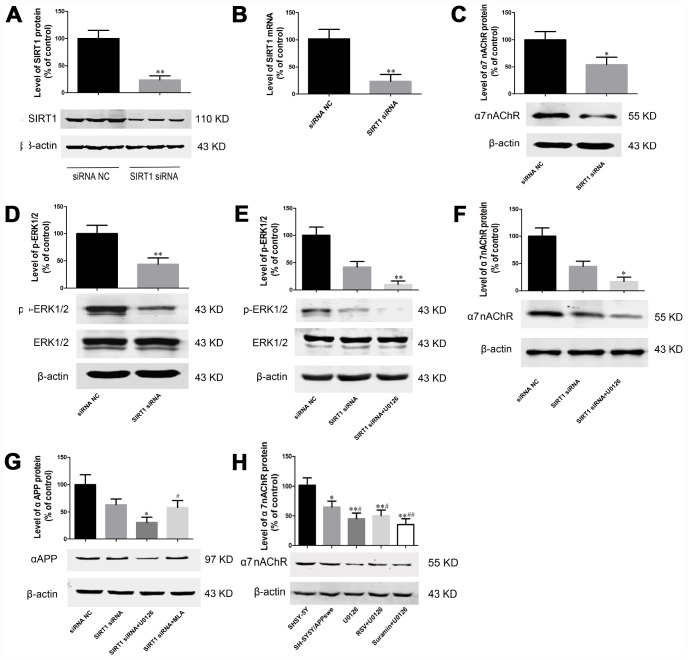
**SIRT1 regulates expression of α7 nAChR and αAPP by SH-SY5Y/APPswe cells through the MAPK pathway.** Transfection with SIRT1 siRNA reduced the level of both SIRT1 protein (**A**) and mRNA (**B**), as determined by western blotting and qRT-PCR, respectively. (**C**) Knock-down of SIRT1 reduced the level of α7 nAChR protein. (**D**) Knock-down of SIRT1 reduced the level of p-ERK1/2 protein. After 24 h of transfection, the cells were treated with 10 μM U0126 for 2 hr and the levels of p-ERK1/2 (**E**), and α7 nAChR (**F**) then determined by Western blotting. (**G**) After 24 h of transfection, the cells were treated with 10μM U0126 or MLA for 2 hr, and the level of the αAPP then determined by Western blotting. (**H**) SH-SY5Y/APPswe cells were treated with 50 μM RSV+ 10 μM U1026 or 300 μg/ml suramin+10 μM U1026, and the level of α7 nAChR expression then determined by Western blotting. The values presented are means ± SD. *P<0.05 and **P<0.01 compared to the negative control group, ^#^*P*<0.05 compared to the group treated with U1026, as determined by analysis of variance (ANOVA), followed by the Tukey HSD test. Representative western blots are shown beneath **A** and **C**–**H**.

Since earlier reports indicate that the ERK1/2 pathway plays an important role in the generation and degradation of the Aβ peptide [31–32], we determined the effects of knock-down SIRT1 in SH-SY5Y/APPswe cells on this pathway. Both qRT-PCR and Western blotting confirmed that transfection with SIRT1-siRNA reduced the levels of SIRT1 protein and mRNA (Figure 6A and 6B). This knock-down also remarkably decreased the levels α7 nAChR and p-ERK1/2 (Figure 6C and 6D). Treatment with an ERK1/2 inhibitor reduced the levels of p-ERK1/2 and α7 nAChR even more than knock-down of SIRT1 alone (Figure 6E, 6F).

### Effects of U0126 and MLA on the expression of α7 nAChR and αAPP and activation of SIRT1 on the expression of α7 nAChR in SH-SY5Y/swe cells

On the other hand, inhibitors of both ERK1/2 and α7 nAChR decreased the expression of αAPP by SH-SY5Y/APPswe cells, more so in the former case (Figure 6G). Furthermore, U0126 alone decreased the expression of α7 nAChR, while RSV attenuated this effect; in addition, suramin together with U1026 reduced the expression of α7 nAChR further (Figure 6H).

### Effects of various compounds on the expression of α7 nAChR, p-ERK1/2, ERK1/2, and SIRT1 by SH-SY5Y cells

**Figure 7:**
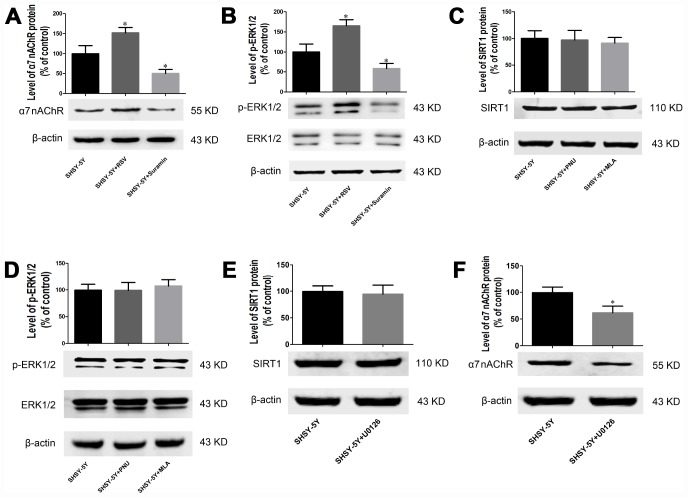
**SIRT1 regulates expression of α7 nAChR and p-ERK1/2 in SH-SY5Y cells through the MAPK pathway.** (**A**) Effects of RSV and suramin on the level of α7 nAChR protein, as determined by Western blotting. (**B**) Effects of RSV and suramin on the level of ERK1/2, as determined by Western blotting. (**C**) Lack of effect of PNU or MLA on the level of SIRT1 protein. (**D**) Lack of effect of PNU or MLA on the level of ERK1/2 protein. (**E**) Lack of effect of U0126 on the level of SIRT1 protein. (**F**) Effect of U0126 on the level of α7 nAChR protein. The values presented are means ± SD. *P<0.05 and **P<0.01 compared to the SIRT1 siRNA negative control cells, ^#^*P*<0.05 compared to treatment with U1026, as determined by analysis of variance (ANOVA), followed by the Tukey HSD test. Representative western blots are shown beneath each bar graph.

Consistent with the findings above, exposure to RSV for 24 h elevated the levels of α7 nAChR and p-ERK1/2 in SH-SY5Y cells and suramin reversed of all these changes (Figure 7A and 7B), while PNU and MLA cause no significant changes (Figure 7C and 7D). Furthermore, exposure to ERK1/2 inhibitor alone, attenuated expression of α7 nAChR (Figure 7E) without altering SIRT1 expression (Figure 7F).

These observations reveal that SIRT1 regulates expression of α7 nAChR at least in part, by activating of the ERK1/2 pathway and thereby to promoting αAPP expression.

## DISCUSSION

For more than 20 years, the amyloid cascade hypothesis, which proposes that deposition of the Aβ peptide in the brain initiates development of AD, has dominated research in this area [33]. Consequently, there has been considerable effort to reduce the production, facilitate the clearance and prevent aggregation of Aβ. However, several Phase III clinical trials based on such efforts have failed [34–36]. Nonetheless, there can be no doubt that either Aβ plays a prominent role in and/ or is at least an important biomarker of the progression of AD.

SIRT1 plays a vital part in the growth and differentiation of neurons, preventing the apoptotic death of these cells by deacetylating p53 and thereby attenuating its activity [37, 38]. Many investigations have shown that activation of SIRT1 exerts antioxidant [39], anti-inflammatory [40], and neuroprotective properties, reducing neuro toxicity [41] and prevent memory loss [42]. In addition, caloric restriction (CR) lowers expression of β-secretase in mice, in part by activating SIRT1, which in turn up-regulates the transcription factor PGC-1α, part of the AMPK-SIRT1-PGC-1α pathway [43].

Here, we shows that activation of SIRT1 improves the capacity of learning and memory in APP/PS1 double-transgenic mice, which reflects an increased level of p-CREB in RSV group and a reduced level of p-CREB in suramin group; whereas, at same time, the suppressed production of Aβ plaques was observed in the hippocampus and temporal cortices of these animals. In addition, we investigated whether synaptic plasticity and inflammation cytokines is responsible for the improvement of learning and memory capacity in APP/PS1 double-transgenic mice. Our current results showed that RSV treatment increased the levels of SYN and SNAP-25 in APP/PS1 mice as compared to APP/PS1 group, while reduced GFAP and Iba-1 in the same animals, suggesting that improvement of synaptic plasticity and the effect of anti-inflammation induced by RSV might improve learning and memory capacity in mice carrying APP/PS1 mutation. In vitro, treatment of SH-SY5Y/APPswe cells with RSV augmented their expression of αAPP. These data indicate a multi-faceted neuroprotective role of SIRT1 in the brain, which is consistent with previous reports concerning Parkinson's disease and stroke [44, 45].

α7 nAChR is a promising and attractive target for treatment of many human diseases. In several types of cancer, up-regulation of this subunit induced by nicotine or cigarette smoke stimulates the synthesis and release of excitatory neurotransmitters and markedly promotes cell invasion, migration and epithelial-to-mesenchymal transition [46–48]. In addition, α7 nAChR is a novel therapeutic target for treatment of inframammary diseases, including atherosclerosis, diabetes, sepsis and arthritis [49–52].

The Aβ42 peptide exhibits high affinity-binding to α7 nAChR [53]. Other evidence indicates that α7 nAChR is involved in memory and cognitive functions and plays a neuroprotective role in AD by influencing the accumulation and oligomerization of Aβ [54, 55]. Activation of α7 nAChR also protects against the toxicity of Aβ via activation of neurotrophic and cell survival mechanisms [56–58]. Interestingly, we detected elevated expression of both α7 nAChR and αAPP upon activation of SIRT1, while inhibition of SIRT1 had the opposite effects. These results indicate that α7 nAChR plays a key molecular role in SIRT1-mediated production of Aβ.

Signaling via the ERK1/2 pathway plays a crucial role in the initiation and regulation of many cellular processes, such as proliferation, survival and apoptosis [59]. Numerous reports have demonstrated a positive correlation between the levels of increased Aβ and ERK activity, suggestive of a link between ERK activation and AD [60–62]. Here, we also observed that activation of SIRT1 enhances the level of p-ERK1/2 in the brain of mice [63]. Meanwhile, the level of α7 nAChR was lowered by treatment with an inhibitor of ERK1/2, as was the level of αAPP by treatment with MLA, an inhibitor of α7 nAChR. These finding indicate that SIRT1 regulates expression of α7 nAChR and αAPP at least in part through the ERK pathway.

In summary, activation of SIRT1 attenuated the neurotoxicity of Aβ, i.e., improved learning and memory and reduced aggregation of Aβ in the brains of mice carrying the APP/PS1 mutation. In contrast, inhibition of SIRT1 promoted such neurotoxicity. In addition, activation of SIRT1 increased the levels of α7 nAChR, αAPP and phosphor-ERK1/2, while inhibition of ERK1/2 counteracted the increase in α7 nAChR caused by RSV. These findings indicate that neuroprotection by SIRT1 may involve increased expression of α7 nAChR, perhaps mediated by the MAPK/ERK1/2 signaling pathway.

## MATERIALS AND METHODS

### Materials

RSV, Suramin, U0126 and the SIRT1 Assay Kit (Sigma-Aldrich, USA); methyllycaconitine (MLA) (Tocris Bioscience, UK); TRIzol reagent (Invitrogen, China), SYBR Green PCR master mix (Applied Biosystems, USA); ECL Plus reagent (Merck Millipore, Germany); the near-infrared amyloid-β fluorescent CRANAD-58 probe (ab146926, Abcam Inc., USA); rabbit monoclonal anti-p-Erk1/2 and rabbit monoclonal anti-Erk1/2 antibodies (4370 and 4695, Cell Signaling Technology (CST) Inc., USA); rabbit monoclonal anti-SIRT1 antibody and anti-SYP (synaptophysin) (ab12193 and ab8049; Abcam Inc., USA); rabbit polyclonal anti-α7 nAChR and mouse monoclonal anti-β-actin antibodies (sc-58607 and sc-376421, Santa Cruz Inc., USA); rabbit polyclonal anti SNAP-25 (synaptosomal-associated protein 25), rabbit polyclonal anti CREB, rabbit polyclonal anti CREB (phosphpo-Ser133), rabbit polyclonal anti GFAP and rabbit polyclonal anti-Iba-1 (ionized calcium binding adaptor molecule 1) antibodies (GTX113839, GTX112846, GTX130379, GTX108711 and GTX100042, Gentex Inc., USA) and horseradish peroxidase-conjugated secondary antibody (7076s and 7074s, CST Inc., USA) were obtained from the sources indicated.

### Mice

B6.Cg-Tg (APPswe, PSEN 1dE9) mice with an 85Dbo/Mmjax background and strain-matched wild-type (WT) mice were purchased from Shanghai Nanfang Biological Technology Development Co., Ltd. APP/PS1 mice were generated and identified as described previously [24], which is a familial model of AD and represents 1% -3% of the disease (96% is sporadic). At four months of age, 27 double-transgenic mice, and 9 age- and gender- matched WT mice were divided randomly into four groups (n=9/per gourp) as follows: the WT group, APP/PS1 group, RSV-treated group and suramin-treated group. The latter two groups were administered 20 mg/kg RSV or suramin once daily for two months, while the animals in other two groups received an equal volume of physiological saline. All animal experimental procedures were performed in accordance with the guidelines of the Animal Ethical and Experimental Committee of Guizhou Medical University.

### The Morris water maze test

The spatial learning and memory of the mice were determined using the Morris water maze test as described previously [64], this test involves finding a submerged escape platform in a circular pool filled with water (25-26°C) rendered opaque (white) with powdered milk. During the familiarization session and acquisition phase (4 trials/day for 4 consecutive days), each mouse was given as long as 60 s to find the hidden platform and then required to remain seated on this platform for 5 s, after which the animal was returned to its home cage. During the retention phase, the platform was removed from the pool and for 60 s the path taken by each mouse was video-filmed to determine the time required to swim to the original position of the platform, as well as the number of passes over and time spent at this position.

### Near-infrared imaging in vivo

Three mice from each group were selected randomly for near-infrared imaging. A solution of CRANAD-58 was prepared freshly in 15% DMSO, 15% cremorphor, and 70% PBS and then allowed to stabilize for 20 min before injecting, each mouse (2.0 mg/kg) via the tail vein. Fluorescent signals (630 nm excitation filters, 750 nm emissions) form brain regions were monitored and 10 and 20 min after injection with the IVIS-Lumina LT Series III imaging system (PerkinElmer) and the data analyzed using Living Image 4.5 software.

### Confocal laser scanning of brain slices

The distribution of fluorescent probes in sections of brain tissue was examined by confocal laser scanning. A 6-μm frozen brain slice from one mouse in each group was incubated with 4% paraformaldehyde for 10 minutes and then washed with PBS three times, followed by washing with dd water, and mounting with VectaShield media. Florescence was observed under a Zeiss LSM 710 confocal microscope (Zeiss, Germany).

### Cell culture and transfection

SH-SY5Y cells stably expressing the human APP gene carrying the Swedish mutation at residues 670/671 (SH-SY5Y/APPswe cells), kindly provided by Professor Weilin Jin of Shanghai Jiao Tong University, were cultured in DMEM medium containing 10% fetal bovine serum in a humidified chamber under 5% CO_2_ at 37 °C. The SIRT1 siRNA and negative control were designed and synthesized at GenePharma Co., Ltd (Shanghai, China). Transfection was performed using lipofectamine 2000 (Life Technologies) in accordance with the manufacturer’s protocol.

Cell cultures were treated with 50 μM RSV (an antagonist of SIRT1) or 300 μg/ml Suramin (an inhibitor of SIRT1) for 24 hr and then harvested for analysis. In other experiments, 10 μM U0126 (an inhibitor of MAPK/ERK) or 10 μm MLA (an antagonist of α7nAChR) was added to the cultures two hr after transfection. The cell harvested at various time-points, and their lysates analyzed.

### RNA isolation and quantitative real-time PCR (qRT-PCR)

Total RNA was isolated using the TRIzol reagent (Invitrogen). qRT-PCR for determination of SIRT1 mRNA was carried out using the SYBR Green PCR master mix (Applied Biosystems). In brief, the 10-μl reaction mixture contained 1 μl first-strand cDNA, 5 μl 2×SYBR Green Master (Rox) Mix, 0.5 μl each of the forward and reverse primers (10 M), 3 μl DNase and RNase-free H2O. The thermal cycling conditions were 2 min at 50°C and 10 min at 95°C, followed by 40 cycles at 95°C for 15 sec and then 1 min at 60°C. The levels of the SIRT1 and β-actin transcripts were calculated as 2-ΔΔCT, where ΔCT represents the difference between the cycle threshold (CT) values for the target gene and β-actin. The primer sequences were as follows: SIRT1 Forward: 5’- TAGCCTTGTC AGATAAGGAAGGA-3’ Reverse: 5’- ACAGCTTC ACAGTCAACTTTGT-3’β-actin Forward: 5’-GGCAT CCTCACCCTGAAGTA-3’Reverse: 5’-TAGCACAG CCTGGATAGCAA-3’

### SIRT1 deacetylase activity

After 24 h of treatment with RSV or suramin, cells were collected and disrupted with a RIPA lysis buffer containing a protease inhibitor mix. 10-μl samples were mixed with 40 μl assay buffer, followed by incubation for 30 min at 37 °C, addition of 5 μl developing solution and incubation for an additional 10 min at 37 °C. The fluorescence signal was detected using a microplate fluorimeter (Tecan, Hillsborough, NC).

### Western blotting

Western blotting was performed as described previously [31]. The proteins were first separated by 10% SDS-PAGE and then blotted onto polyvinylidene difluoride (PVDF) membranes with a transfer unit (Bio-Rad Inc.). For relative quantification of proteins, these membranes were subsequently incubated with antibody against anti-SYP (1:500 dilution), anti-SNAP-25 (1:1000 dilution), anti-CREB (1:1000 dilution), anti-p-CREB (1:1500 dilution), anti-GFAP (1:5000 dilution), anti-Iba-1 (1:1000 dilution), anti-α7 nAChR (1:1000 dilution), anti-SIRT1 (1:1000 dilution), anti-αAPP (6E10, 1:1500 dilution), anti-ERK1/2 (1:1000 dilution) and anti-p-ERK1/2 (1:1000 dilution) or β-actin antibody (1:5000, dilution), at 4°C overnight. After washing, the membranes were incubated with horseradish peroxidase-conjugated secondary antibody (1:5000) for 60 min. Finally, these membranes were incubated in ECL Plus reagent, and the signals thus obtained visualized by exposure to hyper-performance chemiluminescence film for a period of 30 s to 5 min. The protein levels were normalized to that of β-actin.

### Statistical analysis

All values are expressed as means ± SD. All data were analyzed using two-way analysis of variance (ANOVA) or one-way ANOVA, followed by the Tukey HSD test. A p-value of less than 0.05 was considered statistically significant.

### Ethics approval

Animal use for this study was approved by the Ethical Committee of Guizhou Medical University, China (No. 1702110).
